# The association between cardiorespiratory fitness and the incidence of common mental health disorders: A systematic review and meta-analysis

**DOI:** 10.1016/j.jad.2019.07.088

**Published:** 2019-10-01

**Authors:** A. Kandola, G. Ashdown-Franks, B. Stubbs, D.P.J. Osborn, J.F. Hayes

**Affiliations:** a Division of Psychiatry, University College London, 149 Tottenham Court Road, London W1T 7BN, UK; b Department of Psychological Medicine, Institute of Psychiatry, Psychology, and Neuroscience, King's College London, 16 De Crespigny Park, London, SE5 8AB, UK; c Department of Exercise Sciences, University of Toronto, 27 King's College Circle, Toronto, Ontario, ON M5S, Canada; d Physiotherapy Department, South London and Maudsley National Health Services Foundation Trust, London, SE5 8AZ, UK

**Keywords:** Physical activity, Depression, Anxiety, Prevention, Risk factor, Exercise

## Abstract

**Background:**

Physical activity is associated with a lower incidence of common mental health disorder, but less is known about the impact of cardiorespiratory fitness (CRF).

**Methods:**

In this review, we systematically evaluated the relationship between CRF and the incidence of common mental health disorders in prospective cohort studies. We systematically searched six major electronic databases from inception to 23rd of May 2019. We assessed study quality using the Newcastle–Ottawa scale.

**Results:**

We were able to pool the hazard ratios (HRs) and 95% confidence intervals (CIs) of four studies including at least 27,733,154 person-years of data. We found that low CRF (HR = 1.47, [95% CI 1.23 – 1.76] *p* < 0.001 I^2^ = 85.1) and medium CRF (HR = 1.23, [95% CI 1.09 – 1.38] *p* < 0.001 I^2^ = 87.20) CRF are associated with a 47% and 23% greater risk of a common mental health disorders respectively, compared with high CRF. We found evidence to suggest a dose-response relationship between CRF and the risk of common mental health disorders.

**Limitations:**

We were only able to identify a small number of eligible studies from our search and heterogeneity was substantial in the subsequent meta-analysis.

**Conclusions:**

Our findings indicate that there is a longitudinal association between CRF levels and the risk of a common mental health disorder. CRF levels could be useful for identifying and preventing common mental health disorders at a population-level.

## Highlights

• Low and medium CRF are associated with a greater risk of common mental health disorders.
• CRF appears to have a dose-dependent relationship with common mental health disorders.
• CRF could be useful for predicting and preventing common mental health disorders.

## 1 Introduction

Mental illness accounts for around 32.4% of the total number of years lived with a disability worldwide (Vigo et al., 2016) with depression and anxiety disorders being the first and sixth leading causes of disability respectively (WHO, 2018). Depression and anxiety disorders are a group of common mental health disorders that have a debilitating impact on wellbeing and daily functioning (Kessler, 2012). Common mental health disorders are also associated with a greater risk of cardiovascular disease (Batelaan et al., 2016, Correll et al., 2017, Hare et al., 2014) and all-cause mortality (Machado et al., 2018, Walker et al., 2015). Reducing the incidence of common mental health disorders is one strategy for tackling the overall mental health burden worldwide (Cuijpers et al., 2012, Patel et al., 2018). Particular focus is warranted on methods that can simultaneously address the physical health complications with common mental health disorders and reduce the associated mortality gap (Firth et al., 2019).

The results of several meta-analyses suggest that it is possible to reduce the risk of onset of common mental health disorders in people of different ages and varying degrees of risk (Cuijpers et al., 2008, van Zoonen et al., 2014, Werner-Seidler et al., 2017). An important component of developing effective public health strategies and interventions for preventing common mental health disorders is the identification of factors that influence the risk of onset. While there are genetic factors that influence the development of common mental health disorders (Hyde et al., 2016), there is an increasing recognition of the role of environmental and lifestyle risk factors, such as job strain (Fullana et al., 2019, Köhler et al., 2018).

Several meta-analyses of population-based studies have found that low physical activity is associated with a greater incidence of common mental health disorders (Schuch et al., 2018, Schuch et al., 2019; Teychenne et al., 2010, 2015). The results of several randomized control trials (RCTs) indicate that exercise, a subset of physical activity, is effective for reducing common mental health disorders (Cooney et al., 2013; Kvam et al., 2016; Schuch et al., 2016a; Stonerock et al., 2015, Stubbs et al., 2017). These results suggest that physical activity levels may exert a causal influence on common mental health disorders.

Cardiorespiratory fitness (CRF) refers to the capacity of the cardiovascular and respiratory systems to supply oxygen to muscles, and other bodily tissues, during exertion (Blair et al., 1996). Maintaining a medium or high CRF conveys several health benefits including reducing the risk of cardiovascular disease (Blair et al., 1996, DeFina et al., 2015, Kodama et al., 2009, Lee et al., 2010; Myers et al., 2017). CRF is primarily influenced by physical activity levels and could represent an objective surrogate marker of habitual physical activity (Carrick-Ranson et al., 2014). While there have been comparatively few studies attempting to quantify the relationship between CRF and common mental health disorders, there is some preliminary evidence supporting an association. Several population-based studies have found that low CRF is associated with a greater risk of psychological distress (Oliveira et al., 2019), stress (Kettunen et al., 2014), and common mental health (Dishman et al., 2012a, Loprinzi et al., 2017, Suija et al., 2013, Tolmunen et al., 2006).

In a recent 12-week exercise trial, Rahman et al. (2018) found that increase in CRF predicted greater symptom reductions in people with depression, independently of exercise intensity, age and body mass. They also found that improvements in CRF increased the odds of treatment response at follow up (OR = 3.73, 95% CI 1.22–11.43). One systematic review in people with and without depression found a modest correlation between CRF and the severity of depressive symptoms (*CC* = −0.16, 95% CI −0.21—0.10), in 16 RCTs and population-based studies (Papasavvas et al., 2016).

So far, one systematic review has attempted to quantify the relationship between CRF and the incidence of depressive symptoms in the general population (Schuch et al., 2016b). They found that compared with high CRF, people with low CRF had a 76% increase in rate of incident depression (HR = 1.76, 95% CI 1.61–1.91) and people with medium CRF had a 23% increase (HR = 1.23, 95% CI 1.20–1.38). However, this meta-analysis is only based on two studies with high heterogeneity, making it difficult to draw firm conclusions.

To our knowledge, there are currently no systematic reviews that consider the relationship between CRF and anxiety symptoms in the general population. Despite the comorbidity between depression and anxiety disorders (Kessler et al., 2008), there are also no systematic reviews that assess the relationship between CRF and the collective risk of common mental health disorder incidence.

The purpose of this review is to describe and systematically evaluate the relationship between CRF and the incidence of common mental health disorders, at a population-level. Since the previous systematic review on CRF and depression incidence (Schuch et al., 2016b), several relevant population-based studies have been published that may be eligible for inclusion which would increase the statistical power for meta-analysis.

## 2 Methods

The systematic review was conducted in accordance with the PRISMA (Moher et al., 2009) and MOOSE (Stroup et al., 2000) statement and pre-registered on PROSPERO. https://www.crd.york.ac.uk/prospero/display_record.php?RecordID=126059.

### 2.1 Search procedure

One author (AK) conducted searches in Medline, Embase, PsycINFO, PsycARTICLES, CINAHL, and SPORTdiscus from inception to 23rd of May 2019. In the title, abstract, and keyword fields, we used the following search terms: cardiorespiratory fitness OR cardiovascular fitness OR aerobic fitness OR physical fitness OR oxygen uptake OR VO^2^ OR cardiopulmonary fitness OR exercise capacity OR aerobic capacity AND depress* OR anxi* OR panic disorder OR phobia OR agoraphobia. We also conducted supplementary searches using Google Scholar and reference lists of relevant review papers.

### 2.2 Inclusion/exclusion criteria

We included studies which: (1) record CRF using a measure validated against direct measures of oxygen consumption (further details in Section 2.4) (2) have a prospective study design (3) measure depression, or anxiety disorders at the end point of the study using a clinical diagnosis, hospital admission, or a validated self-report scale with a standardized cut off point (4) do not include participants with a prior diagnosis of any psychiatric condition at baseline

We also only considered studies that were published in peer-reviewed journals and in English. In cases where more than one study was conducted in the same cohort, we only included the study that had the most data, i.e. the largest number of person-years.

### 2.3 Outcome

Our outcomes were incidence of any depressive or anxiety disorder as determined by a clinical diagnosis, medical or insurance record, or a validated self-report scale with a standardised cut off point. This included depression, major depressive disorder, dysthymic depression, generalized anxiety disorders, panic disorders, phobias, and social anxiety disorder.

### 2.4 Exposure

Our exposure was CRF measured using any validated method. Gold standard measures of CRF use a maximal exercise test protocol with gas analysis (American College of Sports Medicine, 2013). But these tests are expensive and difficult to administer in population-based cohorts. For this reason we also include other validated measures of CRF, such as time-to-exhaustion protocols that are highly correlated with gas analysis (*r* = 0.92) (Pollock et al., 1982). We also included studies that estimate CRF (eCRF) using algorithms based on heart rate, body composition, gender, age, smoking, and self-report responses on physical activity questions. These eCRF algorithms are validated against direct measures of oxygen consumption (*r* = 0.66 to 0.83) (Jackson et al., 2012; Mailey et al., 2010; Sloan et al., 2013).

### 2.5 Data extraction

In the first stage of study selection, one author (AK) screened title and abstracts of all studies retrieved by the search. To minimize bias, a second author (GAF) screened titles and abstracts from 30% of the search results. After this stage, both authors (AK and GAF) independently carried out full-text screening of studies that potentially met the criteria to be included in our analysis. A third author (BS) was available to review any discrepancies between the two reviewers.

After compiling a final list of studies for inclusion, both authors (AK and GAF) independently extracted data using a pre-specified form. This included information on participant demographics, study design, CRF measurement, mental health assessment, and data relating to the secondary outcomes. For any missing data, we contacted the study authors directly.

### 2.6 Data synthesis and analysis

We conducted a random effects meta-analysis to calculate a pooled hazard ratio with 95% confidence intervals to investigate the relationship between CRF and incident common mental health disorders. We first assessed the relationship between CRF and the combined risk of any common mental health disorder (either depression or an anxiety disorder), before assessing the risk for depression and anxiety individually. Based on methods used in a previous meta-analysis (Schuch et al., 2016b) and how studies in our final list presented their data, we categorised participants into low, medium, and high CRF groups. These groups are based on tertiles defined by the authors of each study, such as grouping by stanine scores. The details of how these groups are derived in each study are outlined in Table 1. Our primary analysis focussed on comparing the risk of people in the lowest CRF group with the highest CRF (reference category), but we also compared people with medium CRF with high CRF. In cases where the study data were presented in a way that was incompatible with our analysis, we contacted authors for more information. We pooled effect size data as hazard ratio's and 95% confidence intervals reported by the authors using Stata 13 (StataCorp, 2013). We used the I-squared (I^2^) statistic to quantify heterogeneity between studies.

**Table 1:** Study characteristics. HR: Hazard Ratio, SHIP: Study of Health in Pomerania, M-CIDI: Munich-Composite International Diagnostic Interview, VO^2^ peak: peak oxygen consumption, APFT: Army Physical Fitness Test, ARMS: Assessment of Recruitment Motivation and Strength, HUNT: Nord-Trøndelag Health Study, HADS: Hospital Anxiety and Depression Scale, ACLS: Aerobics Center Longitudinal Studies, CES-D: Center for Epidemiologic Studies-Depression.

Study	Cohort	N	Mean age at baseline (SD)	Female (%)	Follow-up	Peron-years	Fitness assessment	Fitness grouping	Mental health assessment	Results (95% CIs)			
										Depression	Anxiety	Other	Adjustments
**Aberg et al. (2012****)**	Military conscripts (Sweden)	1117,292	18	0	3–40 years	Not reported	Maximal cycle ergometer with graded test protocol, using heartrate at exhaustion/body mass	Split using stanine scores	Inpatient record	High (ref) vs low HR = 1.80 (1.64–1.99)			Age, calendar year, BMI, region, conscription test centre, parental education
**Baumeister et al. 2017**	SHIP cohort (Germany)	4308	20–79	50.9	1–8 years	Not reported	Maximal cycle ergometer (modified Jones protocol), using gas analysis	No grouping. CRF as a continuous variable.	M-CIDI	Per SD increase in peak VO^2^ RR 0.71 (0.52–0.98)	Per SD increase in peak VO^2^ RR 0.69 (0.50–0.95)	Per SD increase in peak VO^2^ OR 0.45 (0.24–0.84) for combined MDD and anxiety	Age, gender, year of schooling, smoking, alcohol consumption, waist circumference
**Crowley et al. 2015**	Army recruits (US)	300	22 (3.7)	23.3	8 weeks	0	APFT includes measures of CRF and muscular strength	High fitness >=180 out of 300 points on APFT. Low <180 points	CES-D (>=16)	Low (ref) vs high OR 0.40 (0.19–0.84)			Age, gender, ethnicity, education, marital status, family income, army training confidence, army ID, baseline depression, baseline sleep before training
**Gubata et al. (2013****)**	Army recruits (US)	11,369	18	16.8	1 year	11,369	ARMS test including a submaximal Harvard step test and number of push ups in one minute. Only the step test is considered in this study	Two groups based on passing or failing the step test. This is defined as completing the step test for five minutes at a proper pace	Ambulatory encounter	Pass (ref) vs fail unadjusted IRR 1.40 (1.18–1.67) for mood disorders, IRR 1.32 for MDD (0.92–1.89)	Pass (ref) vs fail adjusted IRR 1.57 (1.22–2.01)	Pass (ref) vs fail adjusted IRR 1.36 (1.23–1.49) for any mental health disorder	Gender, smoking, education
**Nyberg et al. (2018****)**	Military conscripts (Sweden)	1109,786	18	0	3–42 years	27,528,903	Maximal cycle ergometer with graded test protocol, using heartrate at exhaustion/body mass	Split using stanine scores	Inpatient record		High (ref) vs low HR = 1.48 (1.36–1.60)		Cognitive performance, BMI, region, year of enlistment
**Shigdel et al. (2019****)**	HUNT cohort in (Norway)	14,020	52.2 (9)	52	11 years	Not reported	Estimated from physical activity questions, age, waist circumference, resting heartrate and gender	Split using quintiles	HADS (>=8)	High (ref) vs low HR = 1.28 (1.02–1.62)	High (ref) vs low HR = 1.04 (0.83–1.30)		Age, gender, marital status, smoking, alcohol intake, education, diabetes, hypertension, HADS score at baseline, limiting long term illness
**Sui et al., 2009**	ACLS cohort (US)	14,343	44.9 (9.7)	22	1–25 years	174,554	Maximal cycle ergometer (Balke protocol), using time to exhaustion	Split using tertiles (bottom 20% = low, middle 40% = medium and top 40% = high)	CES-D (>=16)	High (ref) vs low HR = 1.94 (1.38–2.72)			Age, baseline examination year, survey response year, stressful occupation, smoking, alcohol, BMI, hypertension, diabetes, abnormal exercise ECG

## 3 Results

### 3.1 Search results

**Fig. 1:**
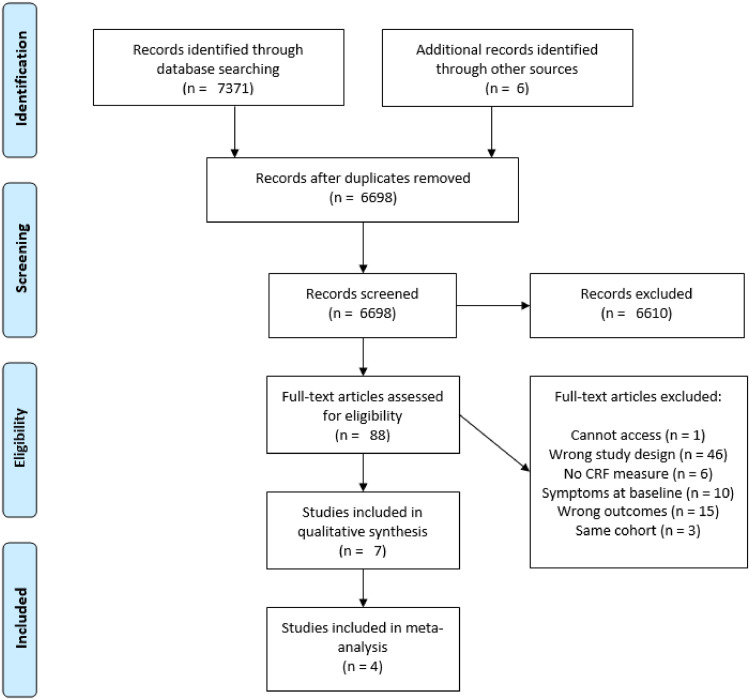
PRISMA flow diagram of study selection.

Our search returned 7371 studies and we identified a further 6 through manual searches of relevant reference lists. After removing duplicates, we screened the titles and abstracts of 6698 studies and excluded 6610. Of the 88 included in our full text screening, 81 were excluded. This left seven studies for the qualitative synthesis and four for the meta-analysis Fig. 1.

We found four eligible studies from the Aerobic Center Longitudinal Studies (ACLS) cohort (Becofsky et al., 2015; Dishman et al., 2012; Sui et al., 2009; Willis et al., 2018). We only included one study, which had the highest number of person-years (Sui et al., 2009).

We also identified two further studies using data from the same registry of Swedish military conscripts (Åberg et al., 2012, Nyberg et al., 2018). In this case, each study investigated different outcomes (depression or anxiety), so both studies are included in the final analysis.

### 3.2 Study characteristics

Individual study characteristics of the seven studies included in this analysis can be found in Table 1.

#### 3.2.1 Participants

In the seven studies selected, there was a total of 1161,632 participants. As there was likely to be significant participant overlap in the two studies using the Swedish military conscript registry, this total only includes one of these studies (Aberg et al., 2012). The percentage of female participants ranged from 0 (Aberg et al., 2012) to 52% (Shigdel et al., 2019), and mean ages range from 18 (Åberg et al., 2012, Gubata et al., 2013) to 52.5 (Shigdel et al., 2019). All studies were in developed, Western countries with most in the US (*n* = 3) and the others in Germany (*n* = 1), Sweden (*n* = 2) and Norway (*n* = 1). Follow-up periods ranged from 8 weeks (Crowley et al., 2015) to 42 years (Nyberg et al., 2018). Of those studies reporting smoking, between 8.8% (Sui et al., 2009) and 29% (Shigdel et al., 2019) of participants reported that they smoked at baseline. According to their BMI, between 17.3% (Gubata et al., 2013) and 26.6% (Shigdel et al., 2019) of participants were obese at baseline.

#### 3.2.2 Fitness assessment

Most of the studies use a maximal exercise test with a cycle ergometer to assess CRF either through measuring heartrate at exhaustion (Åberg et al., 2012, Nyberg et al., 2018) time to exhaustion (Sui et al., 2009) or gas analysis (Baumeister et al., 2017). Shigdel et al. (2019) estimated CRF using an age-adjusted algorithm involving physical activity questions, waist circumference, resting heart-rate and gender. Gubata et al. (2013) used an Assessment of Recruitment Motivation and Strength (ARMS) test involving a five-minute submaximal Harvard step test, and one minute of push ups. Crowley et al. (2015) used the Army Physical Fitness Test (APFT), involving push-ups, sit-ups, and a two mile run to assess CRF and muscular fitness.

Some studies delineate low, medium, and high fitness groups by collating stanine scores (Åberg et al., 2012, Nyberg et al., 2018), tertiles (Sui et al., 2009) or quintiles (Shigdel et al., 2019) from the sample data. One study (Baumeister et al., 2017) did not categorise participants into groups, instead modelling CRF as a continuous outcome. Gubata et al. (2013) split participants into pass/fail groups. The paper defines a ‘pass’ as performing the step test for five minutes at a proper pace and excluded the subsequent push-up test as only 4% of participants failed this part of the test. Crowley et al. (2015) converted raw scores from their APFT into a points-based system with 100 points per test component. High fitness was defined here as >=180 out of 300 points, and low fitness was <180 points. Unlike the other seven studies, fitness here included both CRF and muscular fitness.

#### 3.2.3 Mental health assessment

Some studies assessed mental health using validated scales such as the Hospital Anxiety and Depression Scale (HADS) (Shigdel et al., 2019), Centre for Epidemiologic Studies Depression Scale (CES-D) (Crowley et al., 2015; Sui et al., 2015) and clinical interviews, such as the Munich Composite International Diagnostic Interview (M-CIDI) (Baumeister et al., 2017). The other studies used inpatient, insurance or ambulatory records (Åberg et al., 2012, Gubata et al., 2013, Nyberg et al., 2018). Gubata et al. (2013) included individual categories for mood disorders and major depressive disorder (MDD) using criteria from the Diagnostic and Statistical Manual of Mental Disorders, 4th edition (DSM-4). The mood disorder category includes people with MDD, dysthymia, substance-induced or medically induced mood disorder, adjustment disorder and bipolar disorder.

### 3.3 CRF and common mental health disorders

Of seven studies in the final analysis, it was possible to pool data from four studies, including one study that had outcomes for both anxiety and depression (Shigdel et al., 2019). The pooled analysis was based on data from at least 27,733,154 person-years (to avoid double counting, we calculated person-years without Aberg et al., 2012) and suggested that low CRF is associated with a higher incidence of common mental health disorders compared with high CRF (HR = 1.47, [95% CI 1.23 – 1.76] *p* < 0.001 I^2^ = 85.1). Medium CRF was also associated with a higher incidence of common mental health disorders, compared with high CRF (HR = 1.23, [95% CI 1.09 – 1.38] *p* < 0.001 I^2^ = 87.20). There was substantial heterogeneity in both cases.

**Fig. 2:**
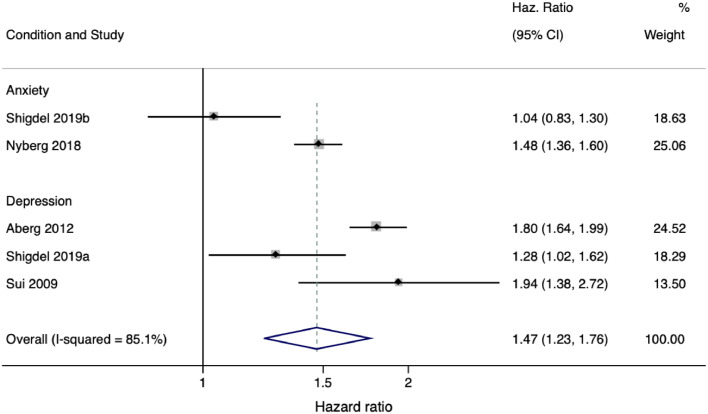
Comparison between low to high CRF for depression or anxiety incidence. One study (Shigdel et al., 2019) included two separate outcomes that are both included in this table, denoted by Shigdel et al., 2019a (depression outcome) and Shigdel 2019b (anxiety outcome).

Baumeister et al. (2017) found that each SD increase in peak VO^2^ is associated with a reduced risk of combined MDD and anxiety incidence (OR = 0.45 [95% CI 0.24–0.84]). This association was stronger than the relationship between CRF and either single diagnosis alone Fig. 2.

### 3.4 CRF and depression

Of seven studies in the final analysis, six included measures of depression (Aberg et al., 2012; Baumeister et al., 2017; Crowley et al., 2015; Gubata et al., 2013, Shigdel et al., 2019; Sui et al., 2009). Data pooled from three of these studies (Åberg et al., 2012, Shigdel et al., 2019; Sui et al., 2009) based on at least 3540,450 person-years demonstrated that low (HR = 1.64, [95% CI 1.29 – 2.08] *p* < 0.001 I^2^ = 73.67) and medium (HR = 1.31 [95% CI 1.10 – 1.55] *p* < 0.01 I^2^ = 77.60) are associated with a greater incidence of depression, compared to high CRF. However, there was substantial heterogeneity.

It was not possible to pool data from the remaining three studies due to different analytical strategies and overlapping participant data. Baumeister et al. (2017) analysed CRF as a continuous variable using Poisson regression and found that each standard deviation increase in CRF (peak VO^2^) reduced the risk of depression by 29% (95% CI 0.52 – 0.98). One study included in the pooled analysis above (Shigdel et al., 2019) also included an analysis of CRF as a continuous variable and detected a significant linear trend (*p* < 0.05) between CRF and incidence depression. Each unit change in metabolic equivalent (MET; a metric for representing unit changes in CRF) being associated with 8% lower risk of depression incidence (OR = 0.92, [95% CI 0.86–0.99]). Another study in the pooled analysis also found a significant linear trend (*p* < 0.001) between CRF group and incidence of depression (Sui et al., 2009).

Crowley et al. (2015) found that participants with low fitness had higher odds of depression (OR 0.40 [95% CI 0.19–0.84]). Using incidence rate data reported in Gubata et al. (2013) we manually calculated unadjusted incidence rate for both mood disorders and MDD. Participants who failed the ARMS test had an increased risk of mood disorders (unadjusted IRR = 1.40, [95% CIs 1.18 – 1.67]) and MDD (IRR = 1.32, [95% CIs 0.92 – 1.89]), but the difference between groups for MDD was not significant. The mood disorder category also included people with bipolar disorder, which was not in our inclusion criteria and not included in our analysis.

### 3.5 CRF and anxiety

Four studies reported anxiety as an outcome measure but only two reported data that could be pooled, which was too few to conduct a meta-analysis.

In three out of four studies that included anxiety as an outcome measure, the results indicate that CRF was associated with a lower risk of anxiety (Baumeister et al., 2017; Gubata et al., 2013, Nyberg et al., 2018, Shigdel et al., 2019). One study including data from 27,528,903 person-years found low CRF (HR 1.48, [95% CI 1.36 – 1.60]) and medium CRF (HR 1.24, [95% CI 1.17 – 1.33]) were associated with a reduction in the risk of anxiety compared to high CRF (Nyberg et al., 2018). Another study found that per standard deviation increase in CRF (measured in METs) there was a 31% decrease in the risk of an anxiety disorder (RR 0.69, [95% CI 0.50 - 0.95]). Gubata et al. (2013 found participants who failed a CRF fitness test had a greater risk of anxiety disorder (adjusted IRR 1.57 [95% CI 1.22 – 2.01]). Finally, one study did not find low CRF (HR 1.04, [95% CI 0.83 – 1.30]) or medium CRF (HR 0.98, [95% CI 0.84 – 1.14]) to be associated with a lower risk of anxiety (Shigdel et al., 2019). This study also analysed CRF as a continuous outcome and found the trend between CRF and anxiety incidence was not significant (*p* = 0.86) and changes in MET did not affect the risk of anxiety incidence (OR = 1.00 [0.94–1.07]).

### 3.6 Study quality

The mean NOS score for all seven studies was 7.57 (range 6 to 9). Five studies were of good quality (Aberg et al., 2012; Baumeister et al., 2017; Nyberg et al., 2018, Shigdel et al., 2019; Sui et al., 2009), one was of fair quality (Crowley et al., 2015) and another of poor quality (Gubata et al., 2013) Table 2.

**Fig. 3:**
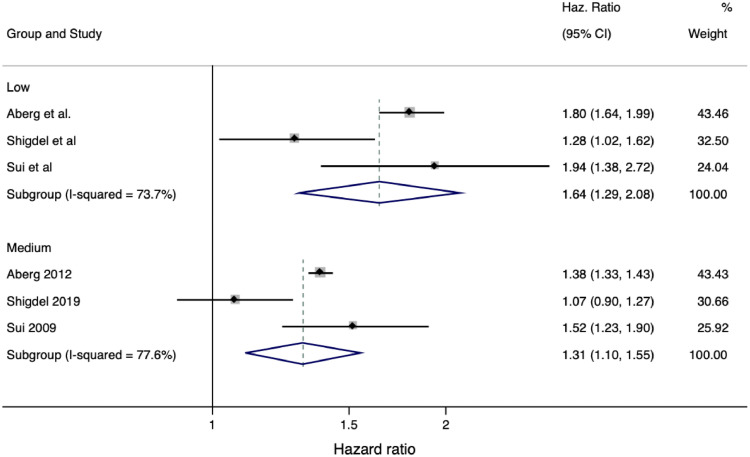
Comparison between low and medium to high CRF for depression incidence.

**Table 2:** Study quality score.

Study	Selection	Comparability	Outcome	Overall
Aberg (2012)	****	**	***	**9**
Baumeister 2017	****	**	**	**8**
Crowley et al., 2015	**	**	**	**6**
Gubata. (2013)	*	**	***	**6**
Nyberg et al. (2018)	****	**	***	**9**
Shigdel et al. (2019)	***	**	**	**7**
Sui et al., 2009	****	**	**	**8**

## 4 Discussion

The aim of this review was to systematically describe and evaluate population-based studies on the relationship between CRF and common mental health disorders. These findings suggest that low and medium CRF are associated with an increased risk of common mental health disorders. Incremental increases in CRF group were associated with proportional decreases in associated risk of new onset common mental health disorders, indicating a dose-response relationship. This was also supported by significant linear trends detected in studies that analysed CRF as a continuous variable (Baumeister et al., 2017; Shigdel et al., 2019) and across the different CRF groups (Sui et al., 2009).

The results of this review are in line with previous meta-analyses (Schuch et al., 2016b) and cross-sectional studies (Loprinzi et al., 2017, Suija et al., 2013, Tolmunen et al., 2006) that have found low CRF is associated with a higher incidence of common mental health disorders. These findings also align with meta-analyses suggesting that low physical activity is associated with a higher risk for common mental health disorders (Schuch et al., 2018, Schuch et al., 2019; Teychenne et al., 2010, 2015).

### 4.1 Possible mechanisms of cardiorespiratory fitness and mental health

The extensive biological impact of exercise on the brain is a possible mechanism underlying this relationship between CRF and common mental health disorders. Exercise and changes in CRF are associated with structural, cellular and molecular changes that promote functioning in brain regions implicated in common mental health disorders (Gujral et al., 2017; Kandola et al., 2016), such as the hippocampus (Firth et al., 2018, Li et al., 2017; Zheng et al., 2019). Exercise and improvements in CRF can reduce inflammation (Eyre et al., 2013, Gleeson et al., 2011, Lavie et al., 2011, Palmefors et al., 2014) and increases resilience to damage from oxidative stress (de Sousa et al., 2017). Both inflammation and oxidative stress are implicated in the pathophysiology of common mental health disorders (Black et al., 2015; Köhler et al., 2017, Kiecolt-Glaser et al., 2015, Moylan et al., 2013). People with low CRF may be forgoing these protective mental health benefits due to a lack of exercise.

### 4.2 The potential importance of cardiorespiratory fitness in mental health

Our findings also highlight a clear need for more population-based studies focussing on CRF. We were only able to identify seven studies for our final analysis, with two from the same cohorts. A recent meta-analysis of physical activity and incidence depression was able to identify 49 unique prospective cohort studies (Schuch et al., 2018). While recording physical activity levels is essential in public health against a backdrop of rising sedentary behaviour, a substantial decline in the CRF of children has been recorded worldwide (Tomkinson et al., 2017). Measures of CRF capture broad physical activity trends with one discrete test using objective, clearly defined markers, such as oxygen consumption. Whereas objective measures of physical activity in field research (e.g. accelerometers) typically record up to a week of data and are poor at capturing non-ambulatory activities, such as cycling or resistance training (Butte et al., 2012, Troiano et al., 2014). While self-report questionnaires are able to capture broader physical activity trends, they are subject to attentional biases (Prince et al., 2008) and correlate poorly with objective measures (*r* = 0.09 to 0.39) (Lee et al., 2011).

CRF measures are not a suitable replacement for physical activity, but they do have complementary benefits that warrant greater efforts to study at a population level. It is also possible that CRF has an independent predictive value from physical activity. In addition to capturing habitual physical activity, CRF also captures the complex interplay between a range of other factors that are relevant in mental health, such as smoking and obesity (DeFina et al., 2015). The value of this is increasingly being recognised in physical health, with several studies finding CRF to be a stronger predictor of cardiovascular disease in population-based studies (Myers et al., 2017; Swift et al., 2013). While no such comparisons exist in mental health, a previous meta-analysis found high activity levels to be associated with a 17% depression incidence compared to low levels based on 1837,794 person-years (Schuch et al., 2018). Whereas in our study, data from 3540,450 person-years indicate a much larger magnitude of effect, with low CRF being associated with a 64% increase in the risk of depression compared to high. While any direct comparison of these studies is difficult, such a difference could indicate that CRF has an independent predictive value for common mental health disorders from physical activity. One study included in this review (Baumeister et al., 2017) included data on physical activity levels as well as CRF. They found that leisure-time, work- and sport-based physical activity was not associated with a significant decrease in the risk of common mental health disorders, whereas each standard deviation increase in CRF (measured using VO2 peak) was associated with a 55% decrease, which was significant.

Greater research in this area is necessary to further explore the role of CRF as a risk factor for common mental health disorders. Such research could inform the development of preventative strategies designed to improve CRF. It is possible to change CRF relatively quickly (Murias et al., 2010), including in people with common mental health conditions (Stubbs et al., 2016). For example, three weekly 45-minute aerobic exercise sessions for three weeks is sufficient to improve CRF by 31% in older people (mean age 68) and 18% in younger people (mean age 23) with further training leading to greater improvements (Murias et al., 2010). Importantly, improving CRF can promote cardiovascular health and reduce the risk of all-cause mortality (Blair et al., 1996, DeFina et al., 2015, Kodama et al., 2009, Lee et al., 2011; Myers et al., 2017). The importance of employing strategies that have dual benefits for physical and mental health in psychiatry is highlighted in a recent Lancet Commission (Firth et al., 2019). There are also several low-cost methods of improving CRF through social prescribing frameworks, such as organised park runs. These properties make CRF a useful public health tool for reducing the incidence of common mental health disorders at a population-level.

These findings may also influence the way exercise-based interventions are administered. For example, there is limited information on the optimal dose of exercise for reducing common mental health symptoms, but some studies suggest that moderate-to-vigorous intensities are most effective (Schuch et al., 2016a, Stanton and Reaburn, 2014). As higher intensity exercise is necessary to influence CRF, it is possible that changes in CRF are an important contributor to the efficacy of exercise treatments. A recent exercise trial found that increases in CRF were significantly associated with reductions in depressive symptoms, independent of the frequency and intensity of the exercise, and strongly predicted treatment response (Rahman et al., 2018). Designing exercise interventions with sufficient intensity, frequency and duration to increase CRF could be one way to promote treatment success. Recording CRF at baseline could be useful in developing exercise protocols that are tailored to individual fitness levels. Measures of CRF could also be used as a tool for monitoring and improving adherence to the intervention. Although it is important to consider that more intense forms of exercise may increase the risk of drop out and recent evidence suggests that low-intensity exercise can still produce similar mental health benefits to higher intensity exercise (Helgadottir et al., 2017).

### 4.3 Strengths and limitations

A strength of our analysis is its inclusion of data from large number of participants with at least 27,733,154 person-years – excluding one (Aberg et al., 2012) of the two studies using Swedish conscript data. The large sample size allows us sufficient power for estimating the relationship between CRF and common mental health disorders at a population level.

But there are also several limitations of this review. We were only able to identify seven studies that met the inclusion criteria for analysis. While participants were from a wide range of ages (18 to 52) and followed for up to 42 years, the small number of studies limited our ability to perform any subgroup analysis. These studies were also all from developed, Western countries, which further limits our ability to generalise these findings. We also detected substantial heterogeneity between studies. Several factors are likely to have contributed to this. The outcome measures varied from self-report questionnaires and clinical interviews to inpatient records. The different outcome measures are likely to capture different populations. For example, self-report measures may capture people with common mental health symptoms who are not receiving treatment, but studies using inpatient records will only include people who are receiving treatment.

Methods for collecting and analysing CRF data also varied across the studies, and may have contributed to the substantial heterogeneity found here. As a result, it was only possible to pool data from four of the seven included studies. Even within the four included studies, three use a maximal exercise test and another uses a non-exercise algorithm to estimate CRF. This algorithm has been shown to have good predictive value of cardiovascular disease and mortality in the same cohort (Nauman et al., 2017, Nes et al., 2014) and the ACLS cohort (Artero et al., 2014). But its predictive value for mental health is unknown. Just one of seven studies measured CRF using the gold standard maximal exercise test with gas analysis (Baumeister et al., 2017). This was not included in the meta-analysis as CRF was analysed as a continuous variable, which is incompatible with other studies in the meta-analysis. With fitness being an inherently continuous outcome, it is possible that categorising CRF into low, medium and high groups inflated findings in previous studies and our results (Royston et al., 2006).

## 5 Conclusion

The results of this systematic review indicate that low and medium CRF levels are associated with a greater risk of common mental health disorders than high CRF. We found evidence of a dose-response relationship between CRF and the associated risk of common mental health disorders. The limited pool of studies identified here also indicate a need for more CRF research at a population-level.

## Declaration of Competing Interest

No authors declare any conflict of interest
